# Supplementary material for: LcSAO1, an Unconventional DOXB Clade 2OGD Enzyme from Ligusticum chuanxiong Catalyzes the Biosynthesis of Plant-Derived Natural Medicine Butylphthalide
Source: Int J Mol Sci. 2023 Dec 13;24(24):17417. doi: 10.3390/ijms242417417 (PMC10743894; doi:10.3390/ijms242417417)
Supplement: Supplementary file 1 [file ijms-24-17417-s001.zip › Supplementary Image.pdf]

Supplementary information

LcSAO1, an unconventional DOXB clade 2OGD enzyme from *Ligusticum chuanxiong* catalyzes the biosynthesis of plant-derived natural medicine butylphthalide

Xueqing Chen[1,†], Xiaopeng Zhang[1,†], Wenkai Sun[1], Zhuangwei Hou[1], Bao Nie[1], Fengjiao Wang[1], Song Yang[1], Shourui Feng[2], Wei Li[1*] and Li Wang[1*]

Figure S1. Differentially expressed Gene analysis.
Figure S2. Functional characterization of recombinant LcSAO1.
Figure S3. Multi-sequence alignment of LcSAO1 and other 2-PP4D proteins.
Figure S4. Multiple sequence alignment of LcSAO1 and other reported 2-OGD proteins.
Figure S5. LcSAO1 shows low structural similarity with other proteins of LcOGD1-4 by previous research.
Figure S6. LcSAO1 shows high structural similarity with other proteins of DOXB family.

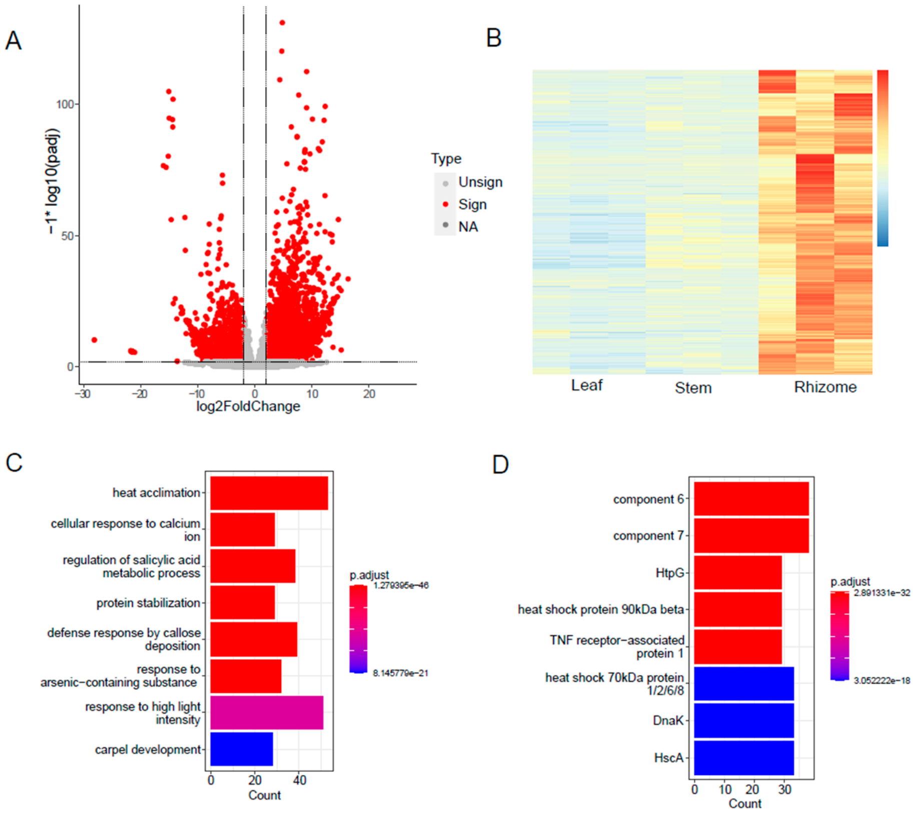

Figure S1. Differentially expressed Gene analysis. (A) Differentially expressed genes in stem and root, the up and down-regulated genes are indicated by red spots. (B) The transcript levels of rhizome-specific differentially expressed genes by FPKM in *L chuanxiong*. (C) Gene ontology (GO) classification of rhizome-specific differentially expressed gene; (D) Kyoto encyclopedia of genes and genomes (KEGG) annotation of rhizome-specific putative protein.

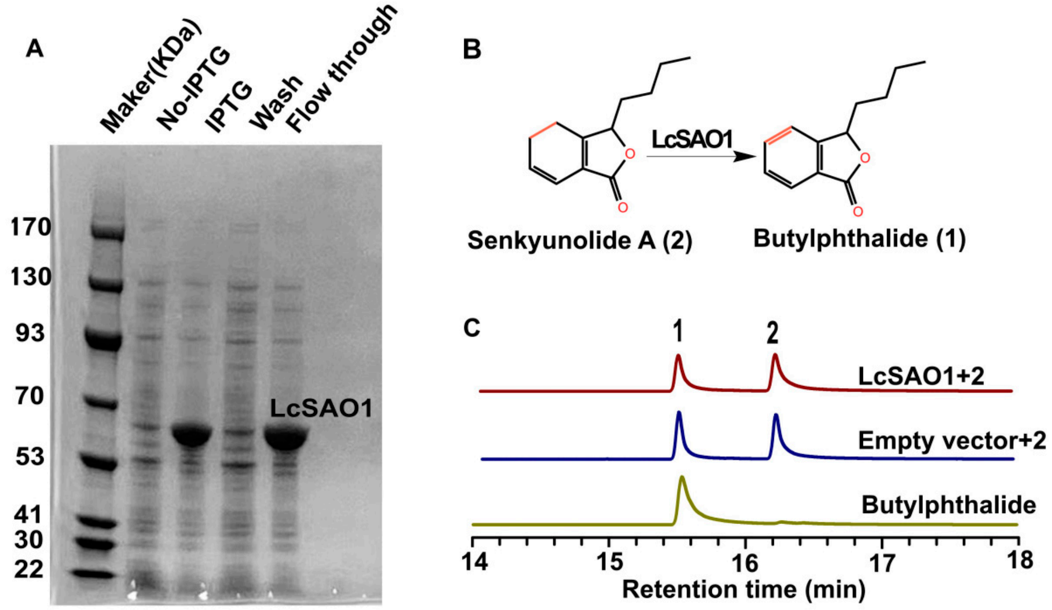

Figure S2. Functional characterization of recombinant LcSAO1. (A) SDS-PAGE of the MBP-tagged LcSAO1 (predicted M.W.: 26.6 kDa). (B) The enzymatic reaction was catalyzed by LcSAO1. (C) GC-MS analysis of **1** (*m/z* 133 to 77) in recombinant protein with **2** to show the specific activity of LcSAO1.

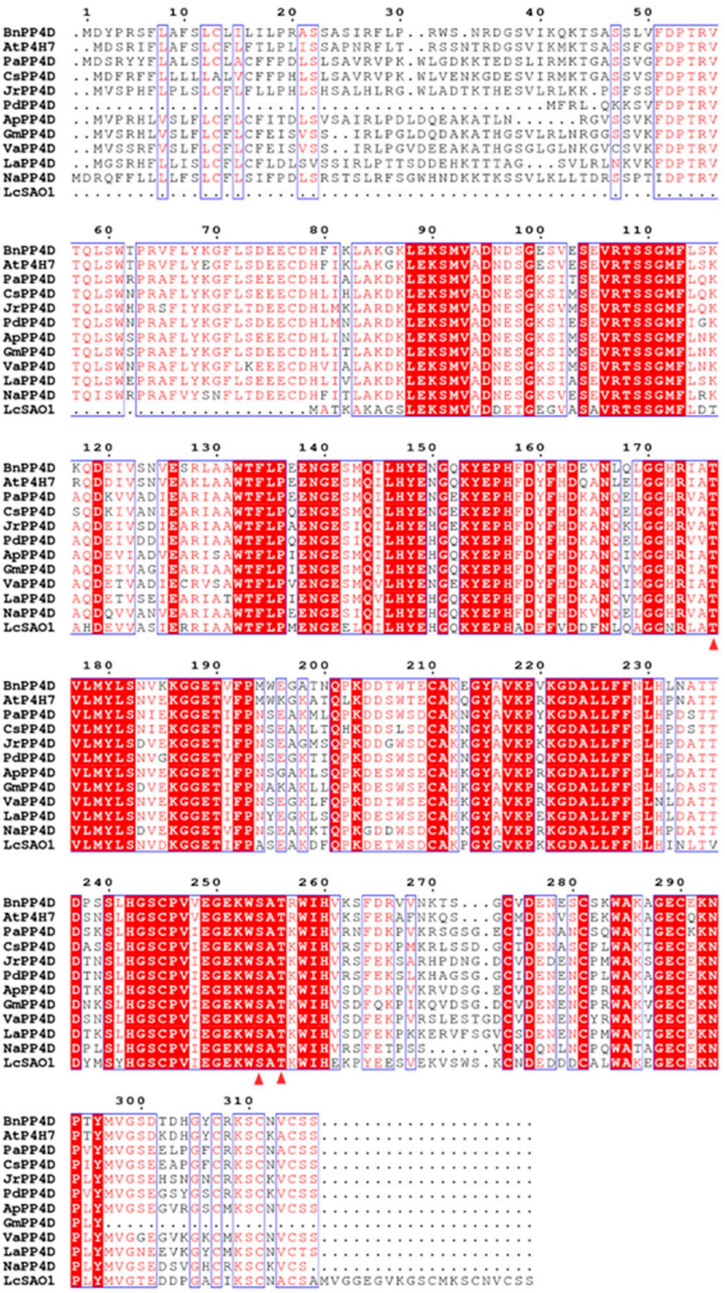

Figure S3. Multi-sequence alignment of LcSAO1 and other 2-PP4D proteins with the conserved domains highlighted. Red triangle indicates the key residue, which can abolish the activity. BnPP4D (*Brassica napus* A0A078JDU9); AtP4H7(*Arabidopsis thaliana*, Q8L970); PaPP4D (*Parasponia andersonii* A0A2P5CEH3); CsPP4D (*Cannabis sativa*, A0A7J6G0Z7), JrPP4D (*Juglans regia*, A0A2I4GLK5), PdPP4D (*Populus deltoides*, A0A8T2WUH2), ApPP4D (*Abrus precatorius*, A0A8B8LV73), GmPP4D (*Glycine max*, I1LWH3), VaPP4D (*Vigna angularis var. angularis* A0A0S3T7C2), LaPP4D (*Lupinus albus* A0A6A4QGV1), NaPP4D (*Nicotiana attenuate*, A0A314L9Z) (Supplementary Table S12).

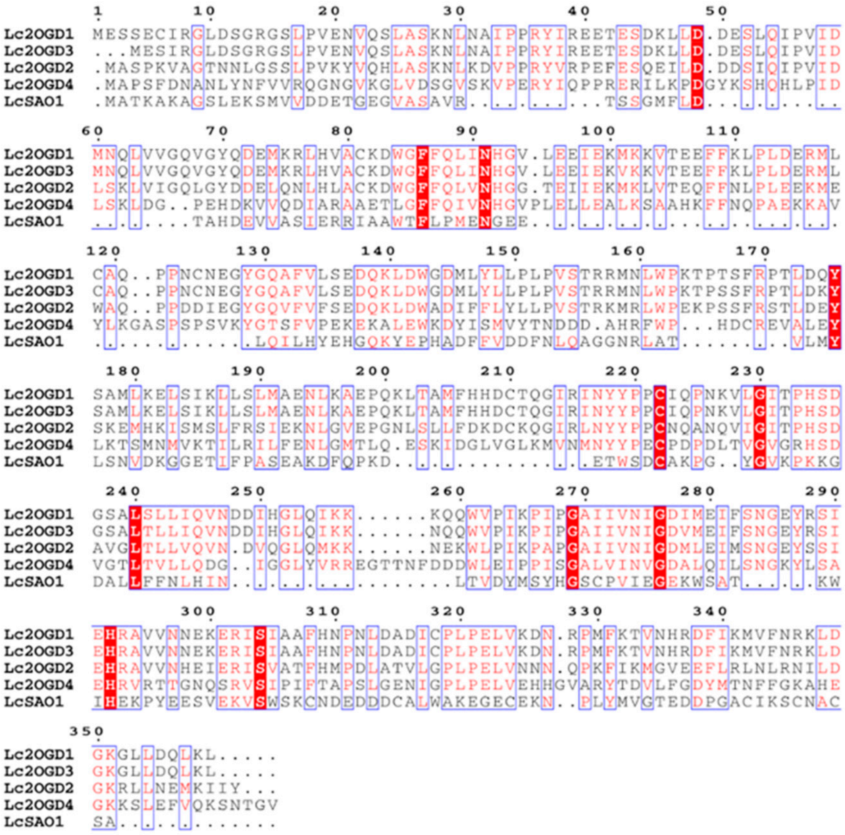

Figure S4. Multiple sequence alignment of LcSAO1 and other reported 2-OGD proteins by Nie et al (Nie et al., 2023). The similarity of LcSAO1 between another four 2OGD proteins ranged from 10.4167% to 12.9167%.

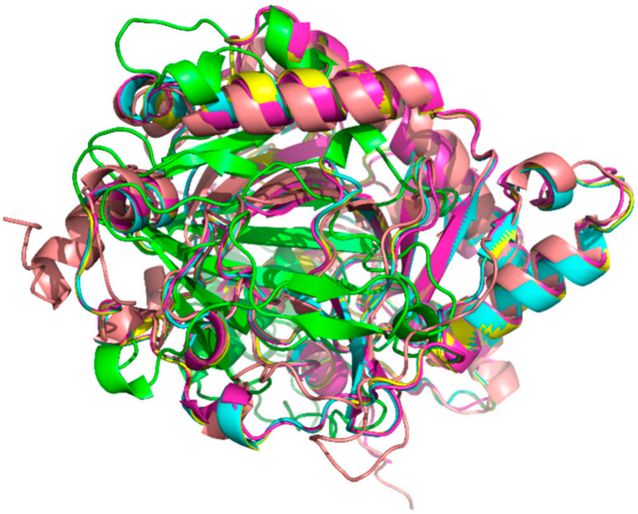

Figure S5. LcSAO1 shows low structural similarity with other proteins of LcOGD1-4 by previous research. (Green: LcSAO1; Blue, LcOGD1; Purple: LcOGD2; Yellow, Lc2OGD3; Pink: LcOGD4, LcOGD1-4 protein sequence was from the paper (Nie et al., 2023)). All the protein structure was predicted by Alpha-Fold2 and presented by pymol (Jumper et al., 2021).

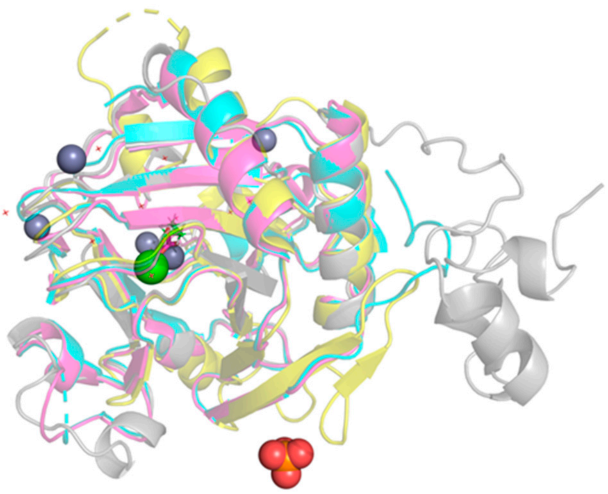

Figure S6. LcSAO1 shows high structural similarity with other proteins of DOXB family. grey: LcSAO1; blue: 2jig; magenta: 3gze; yellow: 3itq
（2jig:https://www.rcsb.org/structure/2JIG;3gze:https://www.rcsb.org/structure/3GZE;
3itq: https://www.rcsb.org/structure/3ITQ)

Nie, B., Chen, X., Hou, Z., Li, C., Sun, W., Ji, J., Zang, L., Yang, S., Fan, P., Zhang, W., Li, H., Tan, Y., Li, W., Wang, L., 2023. Haplotype-phased genome revealed the butylphthalide biosynthesis and hybrid origin of Ligusticum chuanxiong. https://doi.org/10.1101/2023.06.13.544868.
Jumper, J., Evans, R., Pritzel, A., Green, T., Figurnov, M., Ronneberger, O., Tunyasuvunakool, K., Bates, R., Žídek, A., Potapenko, A., 2021. Highly accurate protein structure prediction with AlphaFold. Nature 596, 583–589. https://doi.org/10.1038/s41586-021-03819-2.
